# The Structure of Rhizosphere Fungal Communities of Wild and Domesticated Rice: Changes in Diversity and Co-occurrence Patterns

**DOI:** 10.3389/fmicb.2021.610823

**Published:** 2021-02-04

**Authors:** Jingjing Chang, Yu Sun, Lei Tian, Li Ji, Shasha Luo, Fahad Nasir, Eiko E. Kuramae, Chunjie Tian

**Affiliations:** ^1^Key Laboratory of Mollisols Agroecology, Northeast Institute of Geography and Agroecology, Chinese Academy of Sciences, Changchun, China; ^2^College of Resources and Environment, University of Chinese Academy of Sciences, Beijing, China; ^3^Department of Microbial Ecology, Netherlands Institute of Ecology NIOO-KNAW, Wageningen, Netherlands; ^4^Ecology and Biodiversity, Institute of Environmental Biology, Utrecht University, Utrecht, Netherlands

**Keywords:** domestication, rhizosphere, soil-borne fungi, co-occurrence patterns, arbuscular mycorrhizal fungi

## Abstract

The rhizosphere fungal community affects the ability of crops to acquire nutrients and their susceptibility to pathogen invasion. However, the effects of rice domestication on the diversity and interactions of rhizosphere fungal community still remain largely unknown. Here, internal transcribed spacer amplicon sequencing was used to systematically analyze the structure of rhizosphere fungal communities of wild and domesticated rice. The results showed that domestication increased the alpha diversity indices of the rice rhizosphere fungal community. The changes of alpha diversity index may be associated with the enrichment of *Acremonium*, *Lecythophora*, and other specific rare taxa in the rhizosphere of domesticated rice. The co-occurrence network showed that the complexity of wild rice rhizosphere fungal community was higher than that of the domesticated rice rhizosphere fungal community. Arbuscular mycorrhizal fungi (AMF) and soilborne fungi were positively and negatively correlated with more fungi in the wild rice rhizosphere, respectively. For restructuring the rhizomicrobial community of domesticated crops, we hypothesize that microbes that hold positive connections with AMF and negative connections with soilborne fungi can be used as potential sources for bio-inoculation. Our findings provide a scientific basis for reshaping the structure of rhizomicrobial community and furthermore create potential for novel intelligent and sustainable agricultural solutions.

## Introduction

The rhizosphere is inhabited by the taxonomically structured fungal community that plays an essential role in absorbing nutrients and provides resistance against pathogen invasion and other abiotic stresses associated with their respective host crops (Stringlis et al., 2018; Perez-Jaramillo et al., 2019). For instance, arbuscular mycorrhizal fungi (AMF) can establish mutualistic endosymbiosis with their respective host crop to improve the absorption of mineral nutrients (predominantly phosphate) and enhance the resistance to pathogens, as well as abiotic stresses within the host plant (Akiyama et al., 2005; Luginbuehl et al., 2017; Wang et al., 2017; Jia et al., 2019; Gao et al., 2020). Contrary to AMF, some soilborne fungi are causative factors for creating disease at the crop level (Delgado-Baquerizo et al., 2020). For example, *Passalora rosicola* and *Fusarium oxysporum* in the rhizosphere were associated with common leaf spot and wilt or root rots (Fravel et al., 2003; Feres et al., 2017). As an important feature of the rhizosphere fungal community, the dynamics of fungi–fungi interactions are recognized to carry out important symbiotic microbial functions for maintaining crop health. Characterizations of these interactions have shed new light on the mechanism of pathogen infection and AMF symbiosis (van der Heijden et al., 2008; Agler et al., 2016). However, at the present time, little is known regarding the fungi–fungi interactions within rhizosphere fungal community. As a result, it is of great interest to reveal the mechanism of pathogen infection and AMF symbiosis.

As an important economic crop, rice (*Oryza* species) is the principal food for half of the world’s population (Wang et al., 2018), and rice domestication has been regarded as a critical development in the history of crop domestication. The existing domesticated rice *Oryza sativa* ssp. *japonica*, *O. sativa* ssp. *Indica*, and *Oryza glaberrima* varieties were domesticated from the wild rice species *Oryza rufipogon*, *Oryza nivara*, and *Oryza barthii*, which originated from China, India, and West Africa, respectively (Huang et al., 2012; Wang et al., 2014). The different origins of these materials make the selection highly oriented, and the influence mechanism of habitat factors is also another major factor affecting the genetic context of the germplasms at the gene level. The rhizomicrobial community shares a long history of coevolution with host rice species (Gosling et al., 2006; Kovach et al., 2007; Bin Rahman and Zhang, 2016; Leff et al., 2017; Chang et al., 2021). Studies showed that wild accessions of crops are more tolerant to stress (i.e., disease tolerance and cold tolerance) than domesticated accessions, which may be associated with the interactions among the rhizomicrobial community (Perez-Jaramillo et al., 2017; Choudhary et al., 2018; Tian et al., 2019). Specifically, our team found that the structures of the bacterial and fungal community of wild rice rhizosphere were more stable than those of domesticated rice after the stimulation of exogenous factors to the rhizosphere, such as application of *Magnaporthe grisea* (Shi et al., 2018, 2019; Xu et al., 2019). Recent investigations have addressed the selective effect on the structure of rhizosphere bacterial community during rice domestication. Furthermore, changes of bacterial communities affecting plant growth and development, and further altering plant traits, have been investigated (Mendes et al., 2013; Shenton et al., 2016; Tian et al., 2017). However, little attention has been given to the complicated changes and the interactions among the rhizosphere fungal community during rice domestication.

The major purpose of the current study was to reveal the selective effect of wild and domesticated rice on the structure of the rhizosphere fungal community. Furthermore, we investigated the interactions of fungi–fungi in the wild and domesticated rice rhizosphere, with emphasis on elucidating the interactions between soilborne fungi, AMF, and other fungi community members. We hypothesized that the domestication of rice decreased the cooperative interactions between AMF and other fungi and further limited the beneficial effect to plant growth. To test the hypothesis, we selected rhizosphere soil samples of 12 accessions of wild and domesticated rice to compare the structure of wild and domesticated rice rhizosphere fungal communities using internal transcribed spacer (ITS) amplicon sequencing.

## Materials and Methods

### Plant Materials

Twelve accessions of wild and domesticated rice were used in this study. Wild rice accessions were obtained from the International Rice Research Institute (IRRI), and domesticated rice accessions were obtained from the Philippines and Jiangxi Academy of Agricultural Sciences, China. Five accessions of wild rice included African wild rice (*O. barthii*) IRGC 106238, common wild rice (*O. rufipogon*) IRGC 106286, common wild rice (*O. rufipogon*) IRGC 106452, Indian wild rice (*O. nivara*) IRGC 86655, and Indian wild rice (*O. nivara*) IRGC 88949. Seven accessions of domesticated rice include African domesticated rice No. 2 (*O. glaberrima*), African domesticated rice No. 3 (*O. glaberrima*), African domesticated rice No. 4 (*O. glaberrima*), Asian domesticated rice japonica Jiangxi (*O. sativa* ssp. *japonica*), Asian domesticated rice japonica Daohuaxiang (*O. sativa* ssp. *japonica*), and Asian domesticated rice cultivars indica 106 and Meitezhen (*O. sativa* ssp. *indica*).

### Sample Collection

The experimental plots were located in the rice experimental station of the Chinese Academy of Sciences (18°19′57 N, 109°27′ E) in Sanya, Hainan Province, and were designed in the standard sized plots for rice cultivation. This area is representative of a typical tropical ocean monsoon climate with a mean annual precipitation of 1,347.5 mm and an annual average temperature of 25.7°C. The experiment was arranged in a randomized block design, and there is a 30-cm distance between each of the plots (1 m^2^) to avoid interference. Before experiments were conducted, five soil samples were sampled from 10-cm soil layer of each plot, and the data confirmed soil property homogeneity. The soil texture of the experimental plots was sandy loam. The soil chemical properties of the experimental plots, including pH, soil organic matter, total nitrogen, total phosphorus, available nitrogen, and available phosphorus were 5.22, 25.61 g/kg, 1.54 g/kg, 0.51 g/kg, 162.72 mg/kg, and 20.64 mg/kg, respectively. The samples were collected on November 5–7, 2017, and the experimental plots were kept flooded with a water depth of 10 cm. Wild rice was shown to have longer root length, higher plant height, and better resistance to numerous biotic and abiotic stresses than domesticated rice, such as lodging and drought tolerance (Tian et al., 2018; Nasir et al., 2019; Yu et al., 2020). As comparison, domesticated rice was reported to have high root biomass, more lateral roots, and higher yield than wild rice (Raju et al., 2014; Shi et al., 2019). The trypan blue method was used as described by Phillips and Hayman (1970) to observe the root colonization of the arbuscular mycorrhiza fungi. The rhizosphere samples (soil adhering to root in 1 mm) were collected at the flowering stage of rice under flooding conditions. Five samples from one experimental plot were well mixed as a biological replicate, and there were five replicates for each rice accession, with the exception of common wild rice (*O. rufipogon*) IRGC 106452 which had four replicates. A total of 59 samples were collected, including 35 from domesticated rice and 24 wild rice samples. The loose soil adhering to the roots was shaken off, and the 1-mm root was subsequently immersed in the tube containing 5 ml of sterile water and vortexed to collect the rhizosphere soil that was tightly attached to the root (Edwards et al., 2015). After a short centrifugation at a relative centrifugal force (RCF) of 10,000 *g* for 30 s and removal of the supernatant, each rhizosphere soil sample was stored at −80°C (Edwards et al., 2015).

### DNA Extraction and Amplicon Sequencing

A 0.5-g rhizosphere soil sample was ground into powder in liquid nitrogen, and DNA was extracted from the soil according to the instructions of the Fast DNA SPIN Kit (Catalog No. 6560-220, MP Biomedicals, Germany). DNA concentrations were measured using a NanoDrop 2000 Spectrophotometer (Thermo Fisher Scientific, Waltham, MA, United States). The quality of DNA was evaluated by 1.2% agarose gel electrophoresis, and only DNA exhibiting a clear resolved band was chosen for further sequencing. Fungal ITS1 was amplified by using the primers ITF5F (GGAAGTAAAAGTCGTAACAAGG) and ITS1R (GCTGCGTTCTTCATCGATGC). Sample-specific 7-bp barcodes were incorporated into the primers for multiplex sequencing. The PCR amplicons were purified with Agencourt AMPure Beads (Beckman Coulter, Indianapolis, IN, United States) and subsequently quantified using the PicoGreen dsDNA Assay Kit (Invitrogen, Carlsbad, CA, United States). The 250-bp paired-end sequencing was performed by using the Illumina MiSeq platform. The raw sequencing data were processed by using QIIME v2.0.0 (Quantitative Insights Into Microbial Ecology)^1^ (Caporaso et al., 2012), and paired-end reads were merged by using FLASH v1.2.7 (Magoč and Salzberg, 2011). Sequences shorter than 300 bp, average quality scores of <20, with ambiguous bases, and with mononucleotide repeats of >8 bp were filtered using Trimmomatic v0.33 (Bolger et al., 2014). Chimeric sequences were identified and removed using UCHIME v4.2.0 (Edgar et al., 2011). The remaining high-quality sequences were clustered into operational taxonomic units (OTUs) at a 97% sequence identity by UPARSE v7.0.1001 (Edgar, 2013). A total of 1,689,479 raw fungal reads were obtained, and after quality filtering, a total of 1,687,277 clean reads were obtained. The rarefaction curves already reached a plateau, indicating that the sequencing coverage was valid to quantify the majority of species (Supplementary Figure S1). A representative sequence was selected from each OTU using default parameters. OTU taxonomic classification was conducted by BLAST searching the representative sequence set against and UNITE database (version 5.0)^2^ (Koljalg et al., 2013). Potential plant pathogens for fungal communities and AMF were determined using the FUNGuild database.^3^

### Statistical Analysis

Permutational multivariate analysis of variance (PERMANOVA) was used to detect significant changes in the microbiota structure using pseudo-F value as a proxy for the strength of an individual factor (rice domestication, rice species, or origins) on the fungal community composition (Tkacz et al., 2020). For the PERMANOVA of domestication factor, all samples were separated into two groups: wild and domesticated rice. For the PERMANOVA of accessions factor, all samples were separated into 12 groups of rice species as described in section “Materials and Methods.” For the PERMANOVA of origins factor, all samples were separated into two groups: African rice and Asian rice.

Principal coordinates analysis (PCoA) was used to visualize the difference of fungal classification of wild and domesticated rice rhizosphere based on the Bray–Curtis dissimilarity matrix with the “vegan” package in R (v3.6.1). The statistical significances of the clustering patterns in ordination plots were subsequently evaluated by using PERMANOVA.

Richness (Chao1 index) and evenness (Shannon index) between samples were used for alpha diversity. Chao1 and Shannon indices were calculated by “vegan” and “picante” packages in R (v3.6.1) (Hughes and Hellmann, 2005). To evaluate the difference of Chao1 and Shannon index across wild and domesticated rice rhizospheres, a one-way analysis of variance was conducted with a Tukey’s test (*P* < 0.05) using SPSS v20.0 (IBM, Chicago, IL, United States). To test the number of shared and unique OTUs across wild and domesticated rice rhizosphere, the “VennDiagram” package in R (v3.6.1) was used (Fouts et al., 2012).

To determine the co-occurrence patterns of fungal communities between wild and domesticated rice rhizospheres, we calculated SparCC’s rank correlation coefficients (Python 2.6.1) of taxonomic genera. Here, all samples were grouped into wild and domesticated rice. This method randomly creates 100 simulation datasets from the original data and calculates the pseudo-*P* value by determining how many of the 100 datasets produce the same order of magnitude correlation with the original data (Friedman et al., 2012). For wild rice, 25 replicates were used to calculate the correlation coefficients, while 35 replicates were used for domesticated rice. The network was visualized by Gephi 8.0 [SparCC’s *r* (absolute value) > 0.5, *P* < 0.05].

## Results

### Influence of the Rice Domestication, Species, and Origins on Fungal Community Structure

**FIGURE 1:**
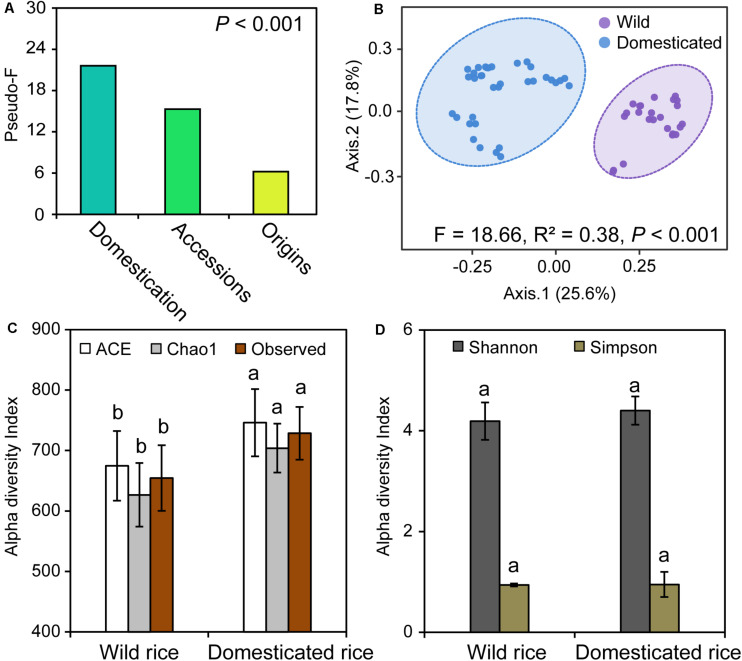
**(A)** The permutational multivariate analysis of variance (PERMANOVA) output showing the importance of rice domestication, rice species, and origins factors shaping the rhizosphere fungal community. **(B)** Compositional changes of fungal communities in the rhizospheres of wild and domesticated rice. **(C,D)** Alpha diversity indices of fungi in the rhizospheres of wild and domesticated rice. The pseudo-F value was used as a proxy for the importance of the factor **(A)**. For the PERMANOVA of domestication factor, all samples were separated into two groups: wild and domesticated rice. For the PERMANOVA of accessions factor, all samples were separated into 12 groups: 12 rice species as described in section “Materials and Methods”. For the PERMANOVA of origins factor, all samples were separated into two groups: African rice and Asian rice. Principal coordinates analysis (PCoA) was performed based on the Bray–Curtis dissimilarity matrix at operational taxonomic units (OTUs) level across all the samples **(B)**. Ellipses demonstrate the mean ± 1 SD, wild rice in purple and domesticated rice in blue. In panels **(C,D)**, wild rice rhizosphere samples, *n* = 24; domesticated rice rhizosphere samples, *n* = 35. Different letters within each column indicated significant differences between wild and domesticated rice based on a one-way ANOVA with a Tukey test at the *P* < 0.05 level.

We initially investigated the structure of fungal communities of 12 rice species, which are divided into two groups, wild rice and domesticated rice, with origins from China, India, and West Africa. PERMANOVA was used to determine the strongest influences on the fungal community. Rice domestication, species, and origins were found to significantly influence the composition of rhizosphere fungal community. Specifically, the strongest factor was rice domestication (pseudo-F: 21.60) (Figure 1A); the second strongest factor was the rice species (pseudo-F: 15.29), and the least important factor was the origins (pseudo-F: 6.22).

### Effect of Domestication on the Composition and Diversity of Fungal Community

To elucidate the effects of rice domestication on the structure of rhizosphere microbiota, PCoA based on Bray–Curtis dissimilarity matrix was performed using 12 accessions that were grouped into wild and domesticated rice. The rhizosphere fungal communities of wild and domesticated rice were significantly different (PERMANOVA, *R*^2^ = 0.28, *P* < 0.001), and the percentages of variation explained by axis 1 and axis 2 were 29.6 and 17.4%, respectively (Figure 1B). To identify the effects of the diversity of the fungal community, we investigated the alpha diversity. Chao1 index, ACE index, and Observed index of the rhizosphere fungal community of domesticated rice were significantly higher than those of wild rice (Figure 1C; *P* < 0.05). There was no significant difference in the Shannon and Simpson indices between wild and domesticated rice (Figure 1D; *P* > 0.05). Additionally, 1,685 shared OTUs accounted for 73% of the total OTUs of wild and domesticated rice rhizosphere (Supplementary Figure S2). The numbers of unique OTUs of fungal communities of wild and domesticated rice rhizospheres were 249 and 373, respectively; accounting for 10.8 and 16.2% of totally wild and domesticated rice OTUs.

**FIGURE 2:**
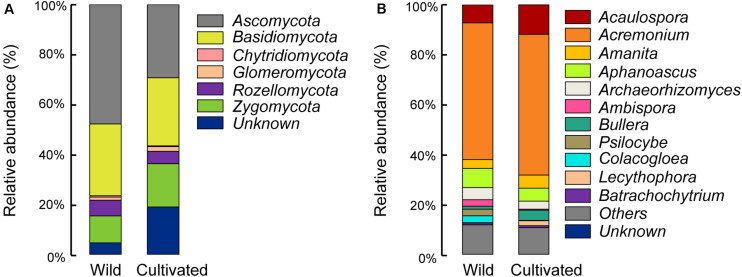
Relative abundances of the dominant fungal phyla **(A)** and fungal genera **(B)** in the rhizospheres of wild and domesticated rice. Relative abundance of the dominant fungal phyla and genera were identified by BLAST searching the representative sequence set against the UNITE database, and the sequences not assigned to kingdom fungi were classified as “unknown.” Treatments included the rhizospheres of wild and domesticated rice. The relative abundance of Zygomycota was highest in bulk soil, while the relative abundance of Ascomycota was highest in the rhizosphere of wild and domesticated rice. The relative abundance of *Acaulospora* was highest in bulk soil, while the relative abundance of *Acremonium* was highest in the rhizosphere of wild and domesticated rice.

**FIGURE 3:**
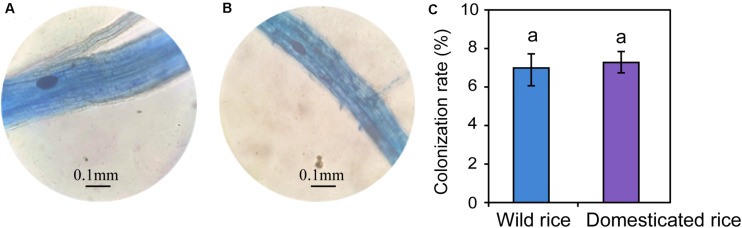
Morphology of the arbuscular mycorrhizal fungi-colonized roots of wild **(A)** and domesticated **(B)** rice. Colonization rate of the arbuscular mycorrhizal fungi in roots of the rice plants **(C)**.

Differences in the main proportion of microbiota were subsequently tested, and the dominant fungal phyla across wild and domesticated rice rhizospheres were Ascomycota, Basidiomycota, and Zygomycota. These data presented relative abundances ranging from 29.32 to 47.79%, 27.24 to 28.70%, 10.81 to 17.32%, respectively (Figure 2A). The relative abundance of Zygomycota in the domesticated rice rhizosphere was higher than that of wild rice. On the other hand, the relative abundances of Ascomycota and Basidiomycota in the rhizosphere of wild rice were higher than that of domesticated rice. AMF correspond to a monophyletic group included in the phylum Glomeromycota, and there was no significant difference in the relative abundance of Glomeromycota between wild and cultivated rice. Results indicated that the AMF were able to colonize both wild and domesticated rice root under flooding conditions (Figures 3A,B). And there was no significant difference between the root colonization of the AMF of wild and domesticated rice (Figure 3C). Further taxonomical classification at the genus level revealed that the relative abundance of *Acremonium* in the rhizosphere of domesticated rice (56.2%) was higher than that of wild rice (54.87%) (Figure 2B).

### Effect of Domestication on the Correlations Between Fungal Communities

**FIGURE 4:**
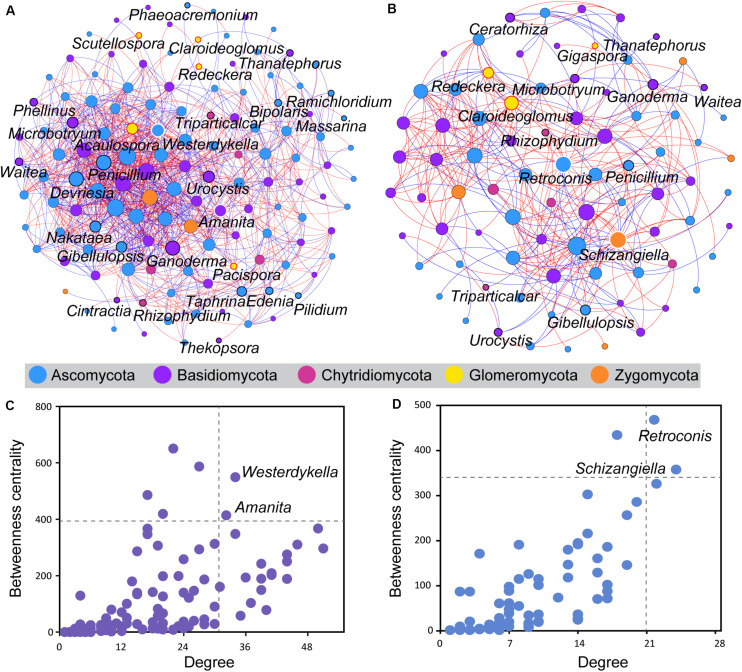
Co-occurrence analysis of the rhizosphere fungal community of wild and domesticated rice. The co-occurrence networks of the rhizosphere fungal community of wild **(A)** and domesticated **(B)** rice were constructed based on the SparCC correlation coefficients. An edge stands for a significant positive correlation (SparCC *r* > 0.5, *P* < 0.05, red line) or significant negative correlation (SparCC *r* < –0.5, *P* < 0.05, blue line). The nodes represent taxa affiliated at the genus level with at least one significant correlation. The size of the node is proportional to the degree (the number of connections). Each node was colored by phyla. The nodes circled by red, white, and black outlines are arbuscular mycorrhizal fungi, hub fungi, and potential plant pathogens, respectively. The nodes representing hub fungi taxa were labeled. The hub taxa of the rhizosphere fungal community of wild **(C)** and domesticated **(D)** rice were nodes showing a high degree and betweenness centrality. The dashed lines indicated the threshold values (top 2%). The nodes representing hub fungi taxa were labeled.

Co-occurrence analysis was used to investigate potential interactions between fungal taxa of wild and domesticated rice rhizospheres (Figure 4). The co-occurrence network of the wild rice rhizosphere consisted of 131 nodes and 1,046 edges, whereas those of domesticated rice consisted of 84 nodes and 346 edges (Supplementary Table S3). Besides, the network of wild rice rhizosphere had more balanced interactions, with 53.92% positive connections and 45.7% negative connections. The discrepancy in the total numbers of correlations among genera in the network input data and that of the co-occurrence network nodes suggests that a tighter association exists among genera in wild rice rhizosphere than what occurs within the domesticated rice rhizosphere.

Five genera of AMF (*Acaulospora*, *Claroideoglomus*, *Pacispora*, *Redeckera*, and *Scutellospora*) were significantly correlated with other fungi in the rhizosphere of wild rice, whereas three groups of AMF (*Claroideoglomus*, *Gigaspora*, and *Redeckera*) were significantly correlated with other fungi in the rhizosphere of domesticated rice. The number of connections of these AMF with other fungi of the wild rice rhizosphere was higher than that of the domesticated rice rhizosphere (Supplementary Table S1). Besides, there are 77% positive connections and 23% negative connections between AMF and other fungi of the wild rice rhizosphere, while the network in the rhizosphere of domesticated rice had 59% positive connections and 41% negative connections between AMF and other fungi. Furthermore, the number of connections between soilborne fungi and other fungi of the wild rice rhizosphere was 327 and higher than that of the domesticated rice rhizosphere (63 connections) (Supplementary Table S1). The network of the wild rice rhizosphere had 53% positive connections and 47% negative connections between soilborne fungi and other fungi. On the other hand, there were 68% positive connections and 32% negative connections between pathogens and other fungi of the domesticated rice rhizosphere.

The higher degree and betweenness centrality of the nodes in the network of the wild rice rhizosphere indicated that the connectivity among nodes is higher in the rhizosphere of wild rice in comparison to that of the domesticated rice rhizosphere. Here, the network hub genera showing a high degree and betweenness centrality were identified. For the wild rice rhizosphere, *Westerdykella* and *Amanita* were defined as the hub taxa and may play an important role in structuring the rhizosphere fungal community (Figure 4C). With the development of domestication, *Retroconis* and *Schizangiella* were defined as the hub taxa in the rhizosphere of domesticated rice (Figure 4D).

## Discussion

Plant genotype, soil, and environmental stresses have been studied intensively to reveal their influences on rhizomicrobiomes (Tkacz et al., 2015; Walitang et al., 2018). However, rhizomicrobiomes are also affected by plant domestication, species, and different origins (Bulgarelli et al., 2015; Leff et al., 2017). The current study was designed and deployed in an effort to determine the role of rice domestication, species, and origins factors in shaping the rhizosphere fungal structure. These efforts ultimately identified rice domestication as the most important factor. Studies on the rhizomicrobiomes of *O. sativa* and their relative wild accessions *O. rufipogon* have shown that the domesticated rice is different from wild rice due to its long domestication history and some distinct rhizomicrobial groups of wild rice that were gradually eliminated (Shenton et al., 2016; Tian et al., 2017; Chang et al., 2021). These observations are in accordance with the hypothesis that the plant host coevolved with their rhizomicrobiomes, and that rhizomicrobial communities are primarily determined by a series of changes due to domestication (Moran and Sloan, 2015; Sasse et al., 2017; Kim et al., 2020).

Rice domestication increased the richness index of the rhizosphere fungal community. There are some lines of experimental evidence demonstrating that the domestication of soybean (*Glycine* ssp.) and corn (*Zea mays* ssp.) also increased the richness index of the rhizosphere bacterial community (Szoboszlay et al., 2015; Chang et al., 2018). One possible explanation for this result might be that, after long-term directional selection, rice domestication resulted in a change of physiological characteristics and affected the levels of metabolites, which may recruit specific fungal species for colonization of the rhizosphere (Grigulis and Clément, 2013). This further proved by our result that *Lecythophora* was the specific fungal genus recruited by domesticated rice rhizosphere in comparison with the wild rice rhizosphere. The relative abundances of *Ambispora* and *Lecythophora* were significantly changed, showing that some microbial groups can be recruited and some microbial groups can be eliminated during rice domestication. Recently, Zhang et al. (2019) found that the duration of rice cultivation affected the alpha diversity of the soil fungal community. These data indicated that the fungal alpha diversity significantly increased when the duration of rice cultivation was less than 15 years and tended to be stable after 15 years. Our study extends this result and demonstrates that domestication leads to the increase of alpha diversity of the rhizosphere fungal community.

Although AMF has been reported to grow under aerobic conditions (Li et al., 2011; Maiti, 2015), we found that the AMF were able to colonize both wild and domesticated rice root under flooding conditions. Recent evidence showed that AMF could colonize rice root grown in flooded conditions, and the rice root colonization by AMF under flooded conditions was lower than that under non-flooded conditions (Hajiboland et al., 2009; Liu et al., 2013, 2014). One possible explanation for this result might be that rice root aerenchyma can transport oxygen to satisfy the aerobic respiration process of the root. Additionally, some of the oxygen will be released into the rhizosphere through the root axis during the transport process to support some members of the rhizomicrobiomes (Colmer, 2003). However, there was no significant difference between the root colonization of AMF between wild and domesticated rice.

It is plausible that the most important contribution of rice domestication might be an alteration in the interactions between microbes. As a consequence, this would further affect the function of the rhizomicrobial community or even the function of the rhizosphere micro-ecosystem. The results of the co-occurrence network-based analysis in this study showed that both node-level and network-level topological features differ between the wild and domesticated rice rhizospheres. The hub taxa of the co-occurrence network of the wild rice rhizosphere were *Westerdykella* and *Amanita*. Additionally, the hub taxa of the co-occurrence network of the domesticated rice rhizosphere were *Retroconis* and *Schizangiella*, which further indicates that the interactions were significantly changed during the domestication of rice. Genera characteristic of the domesticated rice rhizosphere had lower betweenness centrality values and degree values as compared to that of the wild rice rhizosphere. The betweenness centrality represents the importance of the control potential that an individual genus exerts over the interactions of other genera in that network (Greenblum et al., 2012). Additionally, the higher values of connectance, nodes, and correlations made the network of the wild rice rhizosphere more complex than that of domesticated rice rhizosphere. As a result, this enables the wild rice rhizosphere to be more stable against external environmental changes (Wang et al., 2006).

Our team revealed an interesting finding that wild types (including soybean and rice) may have evolved to recruit beneficial microbes in the rhizosphere that are capable of promoting nutrient requisition, biostasis, and disease resistance (Chang et al., 2018; Shi et al., 2019). However, little is known about the interaction of beneficial microbes in the rhizosphere. The interactions between microbes can be the reflection of biological interactions in an ecosystem where species are associated with complicated positive (for example, commensalism and mutualism) and negative (for example, predation and competition) interactions (Faust and Raes, 2012; Jiang et al., 2017). In the current study, the co-occurrence network suggested that AMF of the wild rice rhizosphere are positively correlated with more fungi, while soilborne fungi of the wild rice rhizosphere are negatively correlated with more fungi. Additional commensalism and mutualism connections of AMF with other fungi may result in the characteristics of stress resistance of wild rice. Meanwhile, more and more fungi that compete or predate with soilborne fungi may inhibit the infection of pathogens to the host (Whipps, 2001). This result is in support of the hypothesis that rice domestication decreased the cooperative relationships between AMF and other fungi and further limited the beneficial effect to the growth of plants. Specific microbial groups that keep negative correlations with *Fusarium* spp. have been suggested to be used as candidate biocontrol agents to prevent or control the diseases caused by *Fusarium* (Cobo-Diaz et al., 2019). These findings served as the inspiration for our hypothesis that the microbial groups that hold positive connections with AMF and negative connections with soilborne fungi are likely to be used as potential biocontrol agents to restructure the domesticated rice rhizosphere.

In conclusion, domestication is a very important factor that increased the alpha diversity index (richness index) of the rice rhizosphere fungal community. The co-occurrence patterns demonstrated that the complexity of wild rice rhizosphere was higher than that of domesticated rice, and that AMF and soilborne fungi were positively or negatively correlated with more other fungi, respectively. These results suggested that rice domestication decreased the possibility of benefiting from AMF symbiosis and increased the possibility of infection by pathogens. From the perspective of restructuring the rhizomicrobial community of domesticated crops, we suggest that the microbial groups holding positive connections with AMF and negative connections with plant pathogens can be used as potential biocontrol agents. Our findings are expected to provide rational suggestions for reshaping the structure of rhizomicrobial community of domesticated rice and furthermore highlight the potential to create novel intelligent and sustainable agricultural solutions.

## Data Availability Statement

The datasets presented in this study are deposited at https://www.ncbi.nlm.nih.gov/, SRP298523.

## Author Contributions

CT conceived and designed the study. JC, LJ, and SL helped with the experiment design. FN and LT collected the samples. JC analyzed the data and prepared the figures and table. EK and YS helped with the improvement of the manuscript. All authors read and approved the final manuscript.

## Conflict of Interest

The authors declare that the research was conducted in the absence of any commercial or financial relationships that could be construed as a potential conflict of interest.

## Footnotes

1 http://qiime.org/

2 https://unite.ut.ee/analysis.php/

3 http://www.stbates.org/guilds/app.php
