# Supplementary material for: The Structure of Rhizosphere Fungal Communities of Wild and Domesticated Rice: Changes in Diversity and Co-occurrence Patterns
Source: Front Microbiol. 2021 Feb 4;12:610823. doi: 10.3389/fmicb.2021.610823 (PMC7890246; doi:10.3389/fmicb.2021.610823)
Supplement: Supplementary file 1 [file Data_Sheet_1.docx]

**Supplementary**

**
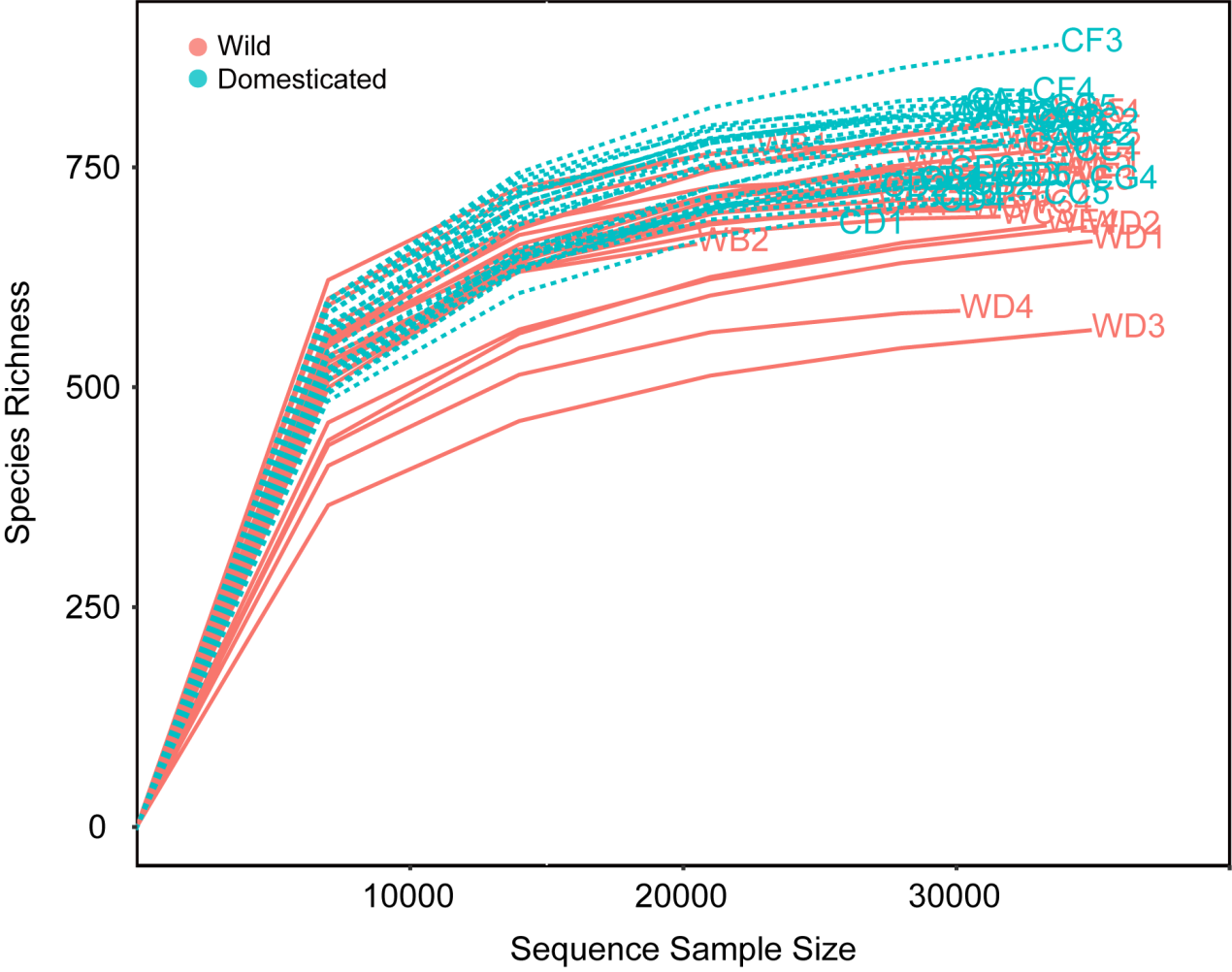
Figure S1.** Rarefaction curves of the fungal reads of wild and domesticated rice rhizosphere. The nodes circled by red, white, and black outlines are arbuscular mycorrhizal fungi, hub fungi and potential plant pathogens, respectively.


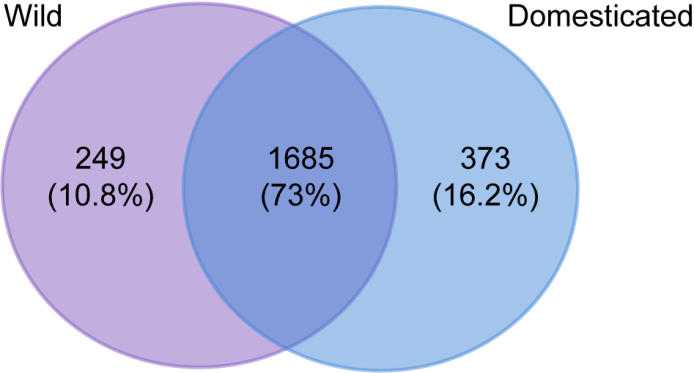


**Figure S2.** Differences of the fungal community OTUs between wild and domesticated rice rhizosphere.Venn diagrams showing the fungal unique and shared OTUs of wild and cultivated rice rhizosphere. The purple circle represents the OTUs distribution of wild rice rhizosphere and the blue circle represents the OTUs distribution of cultivated rice rhizosphere. The number of unique OTUs of wild rice and cultivated rice were 249 and 373, respectively, accounting for 10.8 % and 16.2 % of total wild and cultivated rice OTUs. The number of shared OTUs was 1685, accounting for 73 % of total wild and cultivated rice OTUs.

**Table S1.** Correlations and topological properties of co-occurrence networks of rhizosphere fungal community of wild and domesticated rice

| Network parameters | Wild | Domesticated |
| --- | --- | --- |
| Number of nodes | 131 | 84 |
| Number of nodes (AMF) | 5 | 3 |
| Number of nodes (PPA) | 21 | 10 |
| Number of edges | 1046 | 346 |
| Positive edges | 568 | 222 |
| Negative edges | 478 | 124 |
| Positive edges of AMF | 30 | 16 |
| Negative edges of AMF | 9 | 11 |
| Positive edges of PPA | 171 | 43 |
| Negative edges of PPA | 156 | 20 |
| Average degree | 15.97 | 8.24 |
| Modularity | 0.32 | 0.46 |
| Average path length | 2.44 | 2.72 |
| Connectance | 0.04 | 0.03 |
